# Expectant Parents’ Understanding of the Implications and Management of Fever in the Neonate

**DOI:** 10.1371/journal.pone.0120959

**Published:** 2015-04-08

**Authors:** Sara R. Ahronheim, David McGillivray, Skye Barbic, David Barbic, Stephanie Klam, Paul Brisebois, Kristen Lambrinakos-Raymond, Joe Nemeth

**Affiliations:** 1 Department of Emergency Medicine, Jewish General Hospital, Montreal, Canada; 2 Division of Pediatric Emergency Medicine, Montreal Children's Hospital of the McGill University Hospital Centre, Montreal, Canada; 3 Department of Psychiatry, St Paul’s Hospital, University of British Columbia, Vancouver, Canada; 4 Department of Emergency Medicine, St Paul’s Hospital, University of British Columbia, Vancouver, Canada; 5 Department of Obstetrics and Gynecology, Jewish General Hospital, McGill University, Montreal, Quebec, Canada; 6 McGill University, Montreal, Quebec, Canada; 7 Division of Emergency Medicine, Montreal General Hospital, McGill University Health Center, Montreal, Canada; Université Paris Descartes; AP-HP, Groupe Hospitalier Cochin-Saint-Vincent-de-Paul, FRANCE

## Abstract

**Objective:**

We estimated the extent to which Canadian expectant parents would seek medical care in a febrile neonate (age 30 days or less). We also evaluated expectant parents’ knowledge of signs and symptoms of fever in a neonate, and explored the actions Canadian expectant parents would take to optimize the health of their child.

**Methods:**

We conducted a cross-sectional survey of a sample of expectant parents from a large urban center in Canada. We recruited participants from waiting rooms in an obstetrical ultrasound clinic located in an urban tertiary care hospital in Montreal, Canada. We asked participants nine questions about fever in neonates including if, and how, they would seek care for their neonate if they suspected he/she were febrile.

**Results:**

Among the 355 respondents, (response rate 87%) we found that 75% of parents reported that they would take their febrile neonate for immediate medical assessment, with nearly one fifth of the sample reporting that they would not seek medical care. We found no significant associations between the choice to seek medical care and expectant parents socio-demographic characteristics.

**Conclusions:**

Despite universal access to high quality health care in Canada, our study highlights concerning gaps in the knowledge of the care of the febrile infant in one fifth of expectant parents. Physicians and health providers should strive to provide early education to expectant parents about how to recognize signs of fever in the neonate and how best to seek medical care. This may improve neonatal health outcomes in Canada.

## Introduction

Febrile neonates (aged <30 days) are a high-risk group for serious bacterial infections [1,2]. Reasons for this include susceptibility to infections, difficulty with discriminatory clinical examination, and poor outcomes if not treated or diagnosed promptly [1]. According to a recent World Health Organization update, 1/3 of 4 million annual neonatal deaths worldwide were the result of severe infections [3,4]. Due to the under-recognition of the severity of illness and lack of access to services, most sick neonates in developing countries die before even reaching medical care [5,6]. This is not a problem unique to poor and developing countries. In 2007, among 17 peer developed nations, Canada’s infant mortality rate was second worst [7]. In 2009, the neonatal mortality rate in Canada was 4 per 1000 live births, higher than the 3.6 per 1000 average among 75 high income countries [8]. The provision of high level neonatal and infant care faces specific challenges created by Canada’s geography, low population density and a large proportion of new immigrants from developing countries [9,10]. As a result, prevention of infant and neonatal mortality is now a national policy priority for this country [10,11].

In Canada, a contributing cause of death of neonates is sepsis. Sepsis is defined as the presence (probable or documented) of infection together with systematic manifestations of infection [12]. Fever (defined in this population as a rectal temperature of 38 degrees Celsius or greater [13,14]) can often be the only sign of a serious bacterial infection in the neonate [1,13,15], but may not always be present [13,14]. In the last two decades, clinical guidelines about how to recognize and treat fever in the neonate have been studied, reported, and scrutinized in many journals [16–18]. As well, variations in practice patterns in the evaluation of the febrile newborn have also been observed [19]. Despite ongoing discussions about optimal medical treatment, care cannot commence in this population if the parent does not recognize the fever and seek out medical attention. Consequently, parental understanding of fever is critical.

Remarkably in developed nations such as Canada, very little is known about expectant parents’ knowledge of fever, a potential sign of modifiable and treatable illness in this age group. Recent studies from developed countries have reported that many parents do not know the precise definition of “fever” or believe fever itself is dangerous [20,21]. Expectant parents that are new immigrants, and of lower socioeconomic and educational status, have been shown to possess less knowledge on pediatric fever, and may be less likely to seek care for a febrile neonate [21–25].

To the best of our knowledge, no study has been undertaken to assess Canadian expectant parents’ understanding of fever in the neonate. Thus, the objectives of this study were to estimate the extent to which Canadian expectant parents would seek medical care in a febrile neonate (age 30 days or less), evaluate expectant parents’ knowledge of signs and symptoms of fever in a neonate, and describe the actions Canadian expectant parents would take if they recognized fever in their child.

## Methods

### Setting

In 2011, the population of Canada was 33.4 million persons, with an estimated 378,762 new babies born that year. At this time, Canada had an immigrant population of 6.1 million persons [7], and over 4.7 million persons (14.2% of the population) spoke a language other than English or French most often at home [7]. Montreal is Canada’s second largest city with a population of 1.6 million in 2011 and an area of 4,259 square kilometers. All health care in Canada is delivered through a publically funded health care system, which is mostly free at the point of use.

### Ethics Consideration

This study was reviewed and approved by the institutional research ethics boards at the Jewish General Hospital in Montreal, Canada.

### Study Design, Sample, and Data Collection

We conducted a cross-sectional survey of expectant parents from February to April 2011 in a large Canadian urban setting. We invited English or French speaking expectant parents, aged 18 years and over, to participate in the study. We recruited participants from an obstetrics clinic at the Jewish General Hospital, a tertiary care center serving a diverse patient population in a largely immigrant-based area of Montreal, Quebec, Canada.

We invited expectant parents to our study while they waited for their scheduled obstetrics appointments. If an individual agreed to participate, we asked him/her to provide informed written consent. Once consent was obtained, we asked participants to complete an anonymous survey in either English or French and drop the completed survey in an opaque box. After survey completion, or in the case of participants who did not consent to participate, we gave all individuals an educational pamphlet about the definition and significance of fever in the neonate (see S1 Appendix and S2 Appendix).

### Variables and Measurement

#### Background information

Gender was coded as man or woman. Age was dichotomized into “≤27 years” or “>27 years”. First language spoken at home was coded as “English”, or “French/other”. Stage of pregnancy was coded as “0–26 weeks”, and “27–40 weeks”. Immigration status was coded as “born in Canada” or “moved to Canada”. Education was coded as “high school or less” or “more than high school”. Parent of a second child was coded as “yes” or “no”. Income was coded as “$0-$49,999” or “$50,000+”. All continuous variables were coded based on median values from found in the initial analysis of the data.

#### Knowledge of fever

In order to assess knowledge of fever, we asked expectant parents nine questions (see S3 Appendix and S4 Appendix). Questions were developed based on a review of the literature and consultation with experts in the field of pediatrics, pediatric emergency medicine, and internal medicine. We measured knowledge in three ways. First, we asked one question about whether parents correctly identified the need to obtain medical care in a febrile neonate (scored correct/incorrect). Second, we asked five questions about whether expectant parents could identify potential signs of fever (scored correct/partially correct/incorrect). Finally, we asked three questions to explore the actions the expectant parent would take if they suspected fever in their neonate (see S3 Appendix and S4 Appendix for list of questions).

### Statistical Methods

As mentioned above, we assessed expectant parental knowledge in three ways. First, in order to estimate what proportion of expectant parents would seek medical care in the case they suspect fever in the neonate, we summarized the frequency that parents endorsed one item “*If your baby less than 30 days old has a fever*, *you should bring them to seek medical care immediately*”. We calculated crude odds ratios (OR) and 95% confidence intervals (CI) to ascertain the effects of age, gender, immigration status, education, language spoken, income, stage of pregnancy, and number of other children.

Second, for the five questions asked about potential signs of fever, we summarized the number of parents who answered each question correctly or incorrectly. As we are in the exploratory phase of this research, and do not yet have a psychometrically sound screening tool, we did not add these items to produce a total score. Instead, we counted and displayed the individual responses and displayed the response to each item using frequency tables and cross tabulation.

Finally, for the last three questions about the actions expectant parents would take if they suspected fever in their neonate, we counted the frequency that parents endorsed pre-selected categories for each question.

For all questions in the survey, we also recorded the number of participants who completed each question. In order to test if missing data was missing at random, we performed chi-square tests to test whether there were differences amongst the individuals who completed the items, and those who did not.

All calculations and analyses were performed using SAS software, Version 9.2 (SAS Institute, Inc, Cary, NC).

## Results

**Fig 1:**
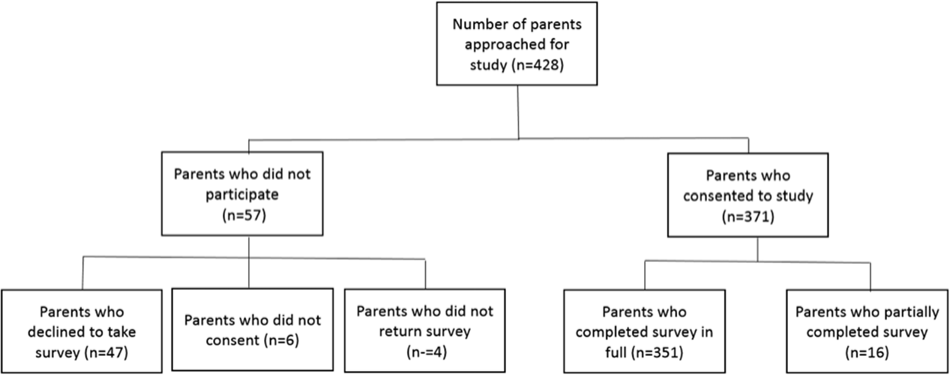
Survey response.

We approached a total of 428 expectant parents for this study. As shown in Fig 1, 371 (87%) agreed to fill in the survey. A total of 377 surveys were partially completed. Twenty three participants (4%) only completed baseline demographic questions, whereas 355 respondents (96%) completed both the demographic and survey parts of the questionnaire. The majority of participants who completed the survey were female (94%), over age 27 (84%), highly educated (60% university or post-graduate degree), and in their second or third trimester of pregnancy (94%). Forty four percent of respondents reported English as their first language, followed by 39% reporting a language other than English or French as their first language, and 17% reporting French as their first language (see Table 1). Individuals who did not fully complete the survey were not statistically different, in terms of demographics, compared to those who fully completed the survey.

**Table 1:** Demographic characteristics of the sample* of people who completed survey questions (n = 355).

Characteristic	N (%)
Age	
<18 years	1 (1)
18–26 years	42 (12)
27–35 years	214 (60)
35+ years	96 (26)
Missing	2 (1)
Female	332(94)
Spoken First Language	
English	156 (44)
French	62 (17)
Other	137 (39)
Education	
High School or less	34 (10)
Community College	76 (21)
University or post-graduate	213 (60)
Missing	32(9)
Ethnicity/Religion	
African American	16 (5)
White	64 (18)
Asian	38 (11)
First Nation	0 (0)
Jewish	60 (17)
Christian	92 (26)
Muslim	57 (16)
Hindu	8 (2)
Other	20 (5)
Immigrant Status	
Born in Canada	158 (45)
Born outside of Canada	197 (55)
Stage of Pregnancy	
1st trimester	15 (4)
2nd trimester	175 (49)
3rd trimester	160 (45)
Missing	5(2)

* In 2011, the population of Canada was 33.4 million persons, with an estimated 378,762 new babies born that year. At this time, Canada had an immigrant population of 6.1 million persons[3], and over 4.7 million persons (14.2% of the population) spoke a language other than English or French most often at home[9]. Montreal is Canada’s second largest city with a population of 1.6 million in 2011 and an area of 4,259 square kilometers. All health care in Canada is delivered through a publically funded health care system, which is mostly free at the point of use.

**Fig 2:**
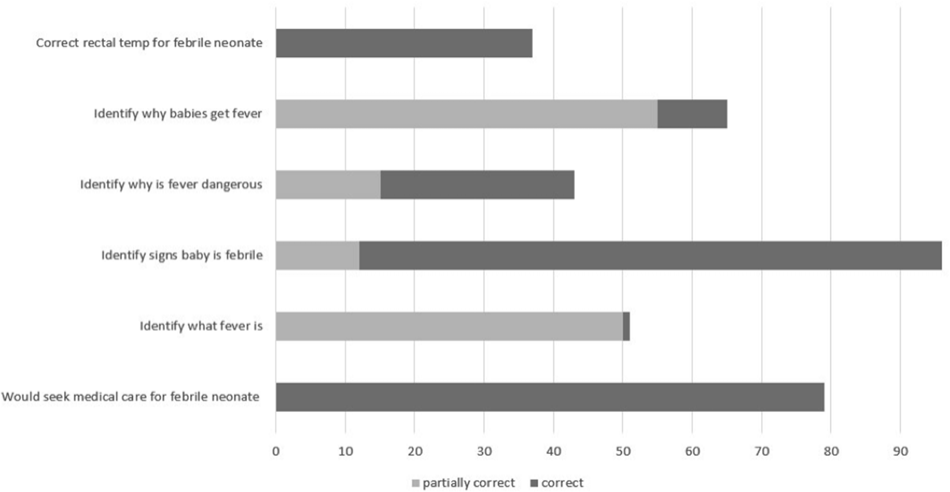
Response pattern of parents to questions about fever (% correct or partially correct).

The first objective was to estimate the extent to which Canadian expectant parents would seek medical care in a febrile neonate (age 30 days or less). Fig 2 summarizes expectant parents’ knowledge of fever. We found that 75% of expectant parents in this study would bring their child to seek medical care if they suspected fever. Nearly one fifth of parents (17%) reported that they would not take their neonate to seek medical care if they suspected a fever. We found that 28 individuals (8%) did not answer this question. As shown in Table 2, we found no significant associations between the choice to seek medical care and any socio-demographic variables.

**Table 2:** Associations between socio-demographics and knowledge of whether to take febrile neonate to seek medical care.

Characteristics	Would seek medical care n = 287/355 (%)	OR (95% CI)
Gender		
Male	21/23 (91.3)	0.42 (0.10–1.75)
Female	266/289 (92.0)	1.0 (ref)
Age		
< 27 years	37/40 (92.5)	0.42 (0.12–1.49)
≥ 27 years	248/310 (80.0)	1.0 (ref)
Education level		
≤ high school	88/103 (85.4)	0.87 (0.56–1.78)
> high school	199/249 (79.9)	1.0 (ref)
Immigration Status		
Born outside of Canada	158/194 (81.4)	1.0 (ref)
Born in Canada	122/149 (81.9)	0.92 (0.51–1.65)
First language		
English	127/152 (83.6)	1.0 (ref)
French or Other	159/198 (80.3)	0.84 (0.60–1.19)
Income*		
≤ $49,999/year	129/154 (83.8)	0.92 (0.69–1.23)
≥ $50,000/year	139/175 (79.4)	1.0 (ref)
Stage of Pregnancy		
≤ 26 weeks	122/149 (81.9)	0.89 (0.67–1.23)
≥ 27 weeks	158/194 (81.4)	1.0 (ref)
Parent of other children		
No	121/149 (81.2)	0.72 (0.81–1.93)
Yes	162/198 (81.8)	1.0 (ref)

* Dollars reported in Canadian Dollars; OR = Odds Ratio, CI- Confidence Interval, Ref = referent group.

The second objective was to evaluate expectant parents’ knowledge of signs and symptoms of fever in a neonate. As shown in Fig 2, we found that most parents were knowledgeable about the signs of illness in the neonate, description of fever, and reasons why fever is dangerous. However, few parents correctly identified the correct rectal temperature (38%) for fever in the neonate.

The third objective was to describe the actions Canadian expectant parents would take if they recognized fever in their child. Table 3 outlines the steps that participants reported they would take if they suspected a fever. Most commonly, parents said that they would call a medical doctor (n = 211, 59.4%), and/or would go to a medical doctor (n = 206, 58%). Approximately one third of the sample reported that they would give their child acetaminophen (n = 118, 33.2%). Amongst participants who reported they would not seek medical care if they suspected a fever (n = 65), 34% (n = 22) reported that they would “relax”, 8% (n = 5) said they do not know what they would do, and 3% said they would rely on personal experience to help their child.

**Table 3:** Actions expectant parents reported that they would take if they suspected fever in their neonate (n = 355).

What is your main source of information about fever in babies less than 30 days old?		If your baby is less than 30 days old and has a fever, what would you do?		Where would you take your baby, less than 30 days old, to seek medical attention when they are ill?	
**Response Categories**	**n**	**Response Categories**	**n**	**Response Categories**	**n**
book given by MD/nurse	102	relax	22	I take care of baby at home	21
book I bought	46	give baby sponge bath	61	I call Info-Sante*	91
magazine or newspaper	13	acetaminophen	118	Clinic	46
internet	19	call MD	211	My pediatrician or family MD	230
nurse/physician	150	go to MD	206	Hospital emergency room	138
family	65	ask friend for help family	42	Other practitioner healer, acupuncturist, etc)	10
		Ask family	393		
		other	3		

*Info-Santé is a Quebec health information phone hotline; MD = medical doctor.

Participants reported accessing varying sources about fever in the neonate. Primarily, expectant parents would seek information from health care professionals, or from books or pamphlets given to them by a nurse or doctor (n = 252, 71%). Participants reported that the Internet (n = 91, 25.6%) and a family member (n = 65, 18.3%) would be other sources of information to learn about how to manage fever in the neonate.

## Discussion

Our study evaluated Canadian expectant parents’ knowledge of fever in the neonate. The main finding of this research was that nearly a fifth of expectant parents in the study reported that they would not take their neonate to seek medical care if they suspected a fever. No demographic variables predicted if expectant parents would seek care for a febrile neonate. Though we found that although most parents were able to identify the signs of a fever and why it may be dangerous, few parents were able to correctly identify the temperature at which a neonate is considered febrile. Although neonatal mortality is less common in Canada compared to developing countries, our findings suggest that further investigation is needed to understand the barriers to knowledge about fever in the neonate in this population.

Most studies to date have examined this topic in populations of parents living in developing countries, summarizing a common need to enhance education about fever in mothers in antenatal care as well as those discharged from health facilities after delivery [6,26,27]. Despite living in a country that offers free universal health care, our study shows that a significant portion of Canadian expectant parents would not consult a physician if they suspected fever in their neonate. This raises questions about pre and postnatal education about fever (or neonatal health in general) in this country. Understanding reasons for these deviations is critical and could shed light how to optimize health outcomes in young infants.

In our study, although immigration status was not a predictor of seeking medical care, we found a significant portion of participants were foreign born immigrants (55%). This was three times greater than the national average of 18% [7]. Efforts to develop any pre/postnatal education materials should carefully consider the cultural backgrounds and norms for the varying groups of individuals who receive health services in countries with a diverse immigrant population such as Canada [28–30]. This is supported by previous studies on this topic [1,2,6,17]. Based on the results of our study, in order to refine current health promotion programs targeted at Canadian expectant parents, future qualitative work is needed to understand the potentially diverse educational needs around this topic [31,32]. As well, prior studies on this topic have been conducted in countries with high population density and smaller geographical challenges than Canada, and less cultural diversity [1,2,6]. Thus, future qualitative efforts will need to consider the unique geographical challenges inherent in Canadian health care.

Finally, we found that most expectant parents reported that they would seek information about fever in the neonate from a medical doctor or a nurse (see Table 3). Even in today’s technological era, only 25% of the sample identified that they would rely on the Internet to query how to treat fever in a neonate. Our study suggest that expectant parents feel that the role of the health care professional in educating around this topic is very important. If not already part of practice, health care professionals involved in prenatal care should provide education about this topic in appointments before and after the delivery. Further, collaboration between the professional societies of family physicians, paediatricians, obstetricians, and nursing is essential to promote early, frequent, and accurate education to expectant parents about this information [33].

### Limitations of Study

Our study was not without limitations. First, given that the sample came from only one Canadian city, it may not be fully representative of the Canadian population. Specifically, our study lacked younger parents (aged 26 years or less), First Nations (aboriginal peoples of Canada) representation, and parents with a low education. It is hard to know exactly how this would have influenced the results; however, we postulate that a population of younger, less experienced, less educated, marginalized parents may have had less knowledge around fever in the neonate. Second, our study was conducted in a single-center. Although the site chosen was quite ethnically and economically diverse, we recognize that a similar study conducted in other large Canadian urban hospitals may give different results. Third, we used a newly developed survey in this study. Further development and refinement of the survey is needed to justify content validity, reliability, and addition of items to produce a total score. Specifically, we acknowledge that future studies that survey expectant parental knowledge of fever in the neonate should include questions that capture other danger signs of severe illness such as those outlined by the World Health Organization in the Integrated Management of Newborn and Childhood Illness guidelines [3]: 1) history of difficulty feeding, 2) movement only when stimulated, 3) temperature below 35.5 degrees Celsius, 5) temperature above 37.5 degrees Celsius, 5) respiratory rate of over 60 breaths per minute, 6) severe chest in drawings, and 7) history of convulsions. In addition, the statement, “If your baby seems unwell, check a rectal temperature” should be used in future assessments of expectant parents’ knowledge of fever. Accurate and timely parental assessment of fever in the neonate, and continued heightened awareness around the topic, may optimize health outcomes of neonates globally.

## Supporting Information

S1 Appendix Information for participants page 1. (TIFF)

S2 Appendix Information for participants page 2. (TIFF)

S3 Appendix Study survey page 1. (TIFF)

S4 Appendix Study survey page 2. (TIF)
