# Supplementary figures and images for: Expectant Parents’ Understanding of the Implications and Management of Fever in the Neonate
Source: PLoS One. 2015 Apr 8;10(4):e0120959. doi: 10.1371/journal.pone.0120959 (PMC4390280; doi:10.1371/journal.pone.0120959)

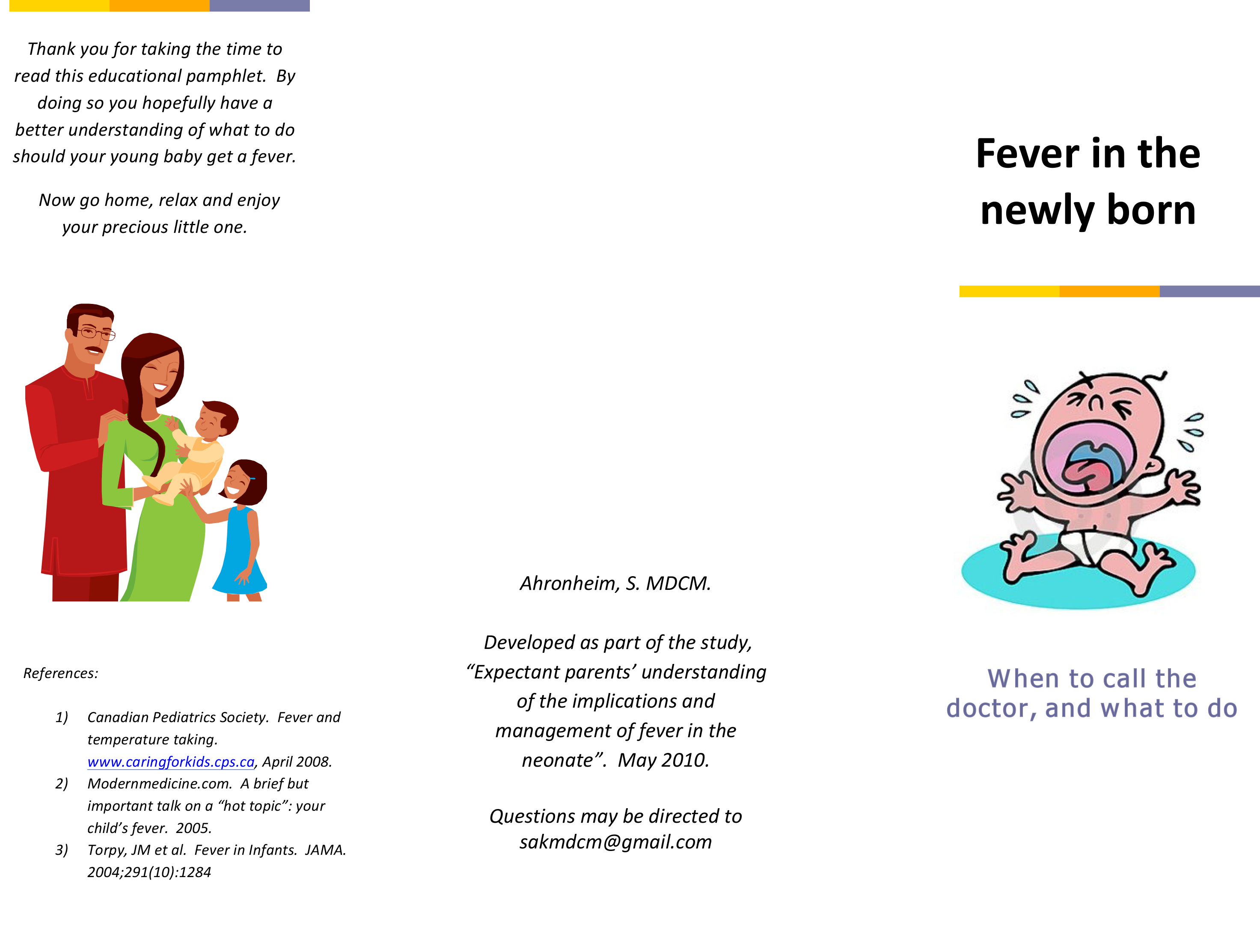

Supplement: S1 Appendix — (TIFF) [file pone.0120959.s001.tiff]

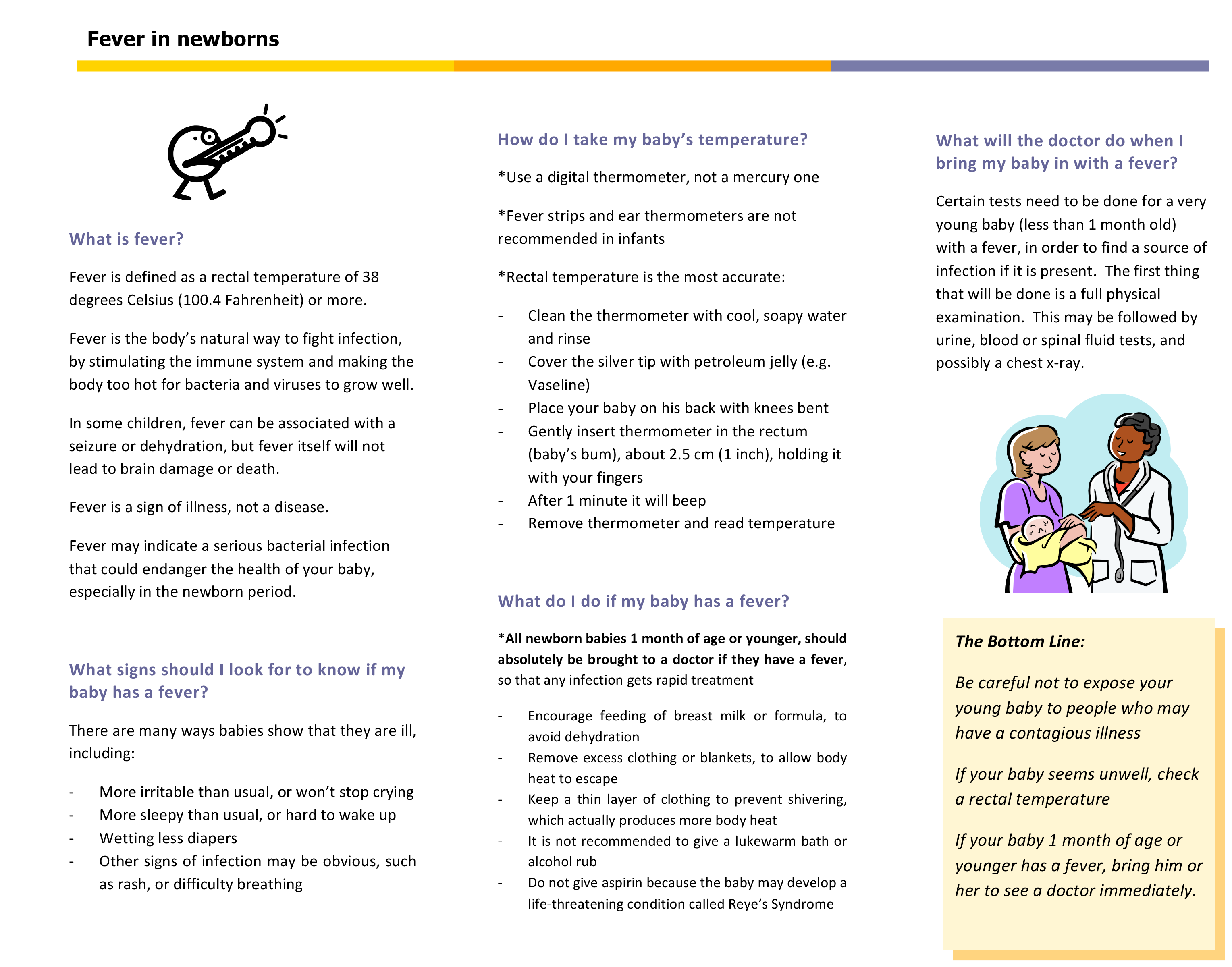

Supplement: S2 Appendix — (TIFF) [file pone.0120959.s002.tiff]

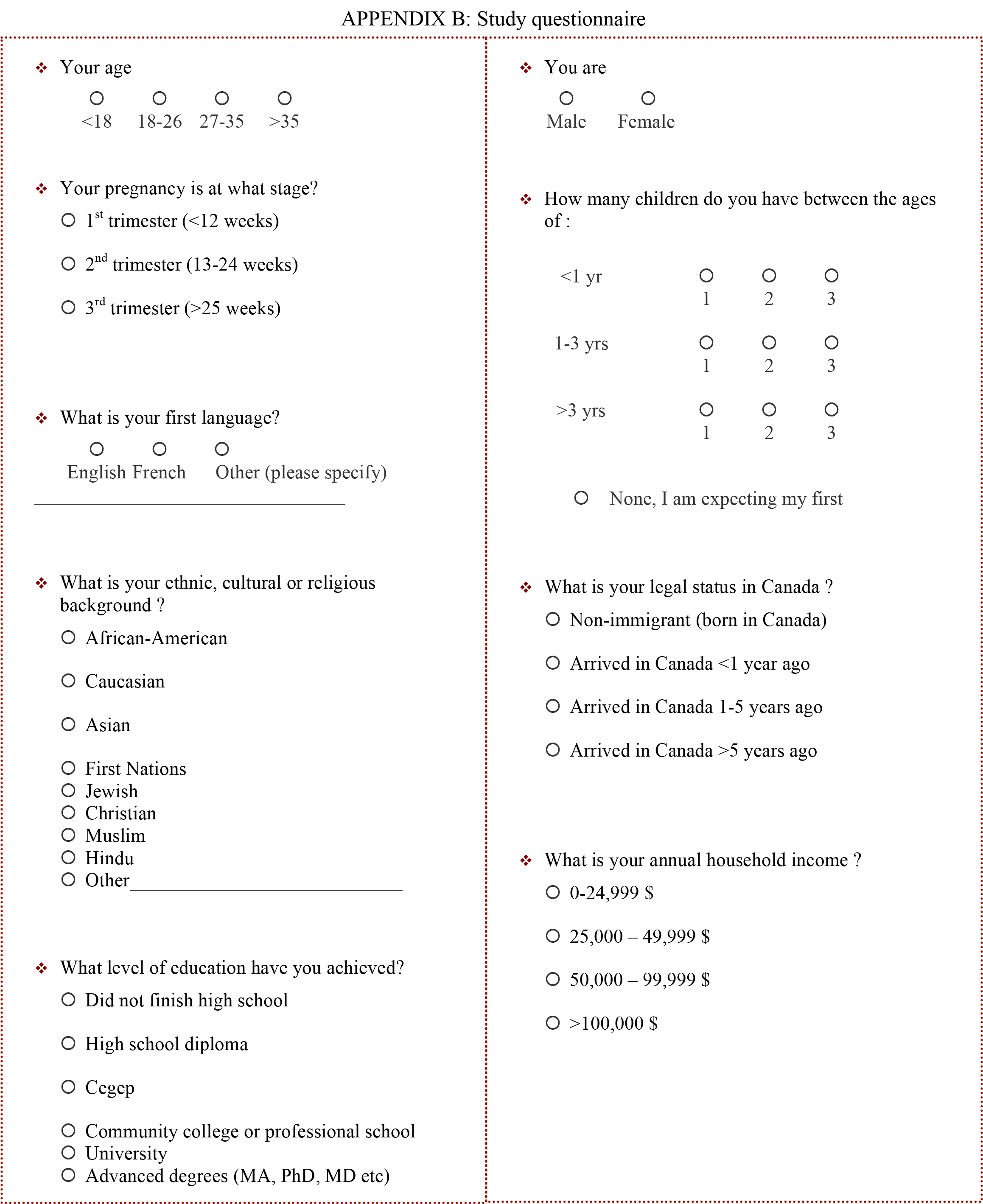

Supplement: S3 Appendix — (TIFF) [file pone.0120959.s003.tiff]

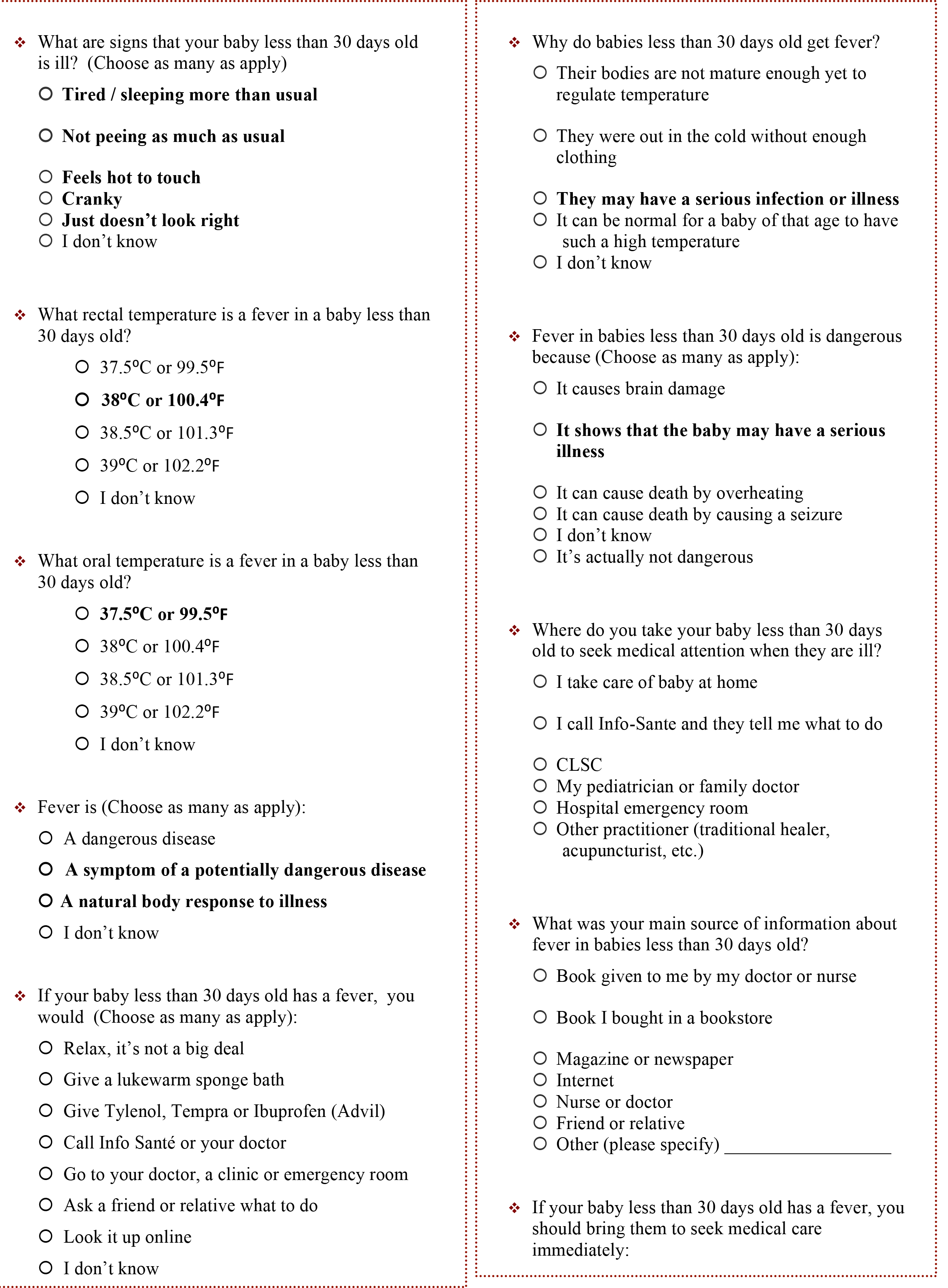

Supplement: S4 Appendix — (TIF) [file pone.0120959.s004.tif]
